# Supplementary material for: Dietary Supplementation with 20-Hydroxyecdysone Ameliorates Hepatic Steatosis and Reduces White Adipose Tissue Mass in Ovariectomized Rats Fed a High-Fat, High-Fructose Diet
Source: Biomedicines. 2023 Jul 23;11(7):2071. doi: 10.3390/biomedicines11072071 (PMC10377470; doi:10.3390/biomedicines11072071)

Supplementary Figure S1

Non-processed Western blot images shown in Figure 3A
The dot box outlines the area presented in the Figure 3A

C-digit-Blot-liver-AMPK

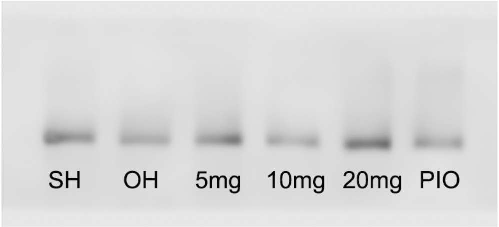

C-digit-Blot-liver-pAMPK-Thr172

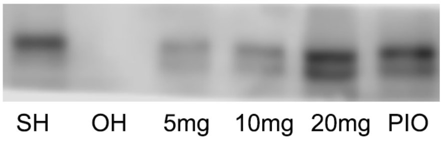

C-digit-Blot-liver-GAPDH

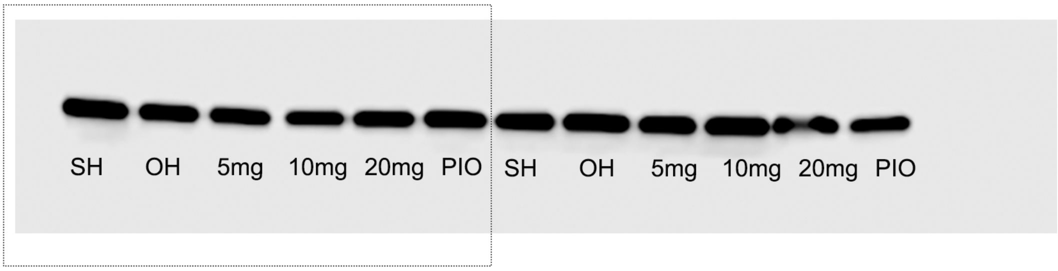

Supplementary Figure S2

Non-processed Western blot images shown in Figure 4A
The dot box outlines the area presented in the Figure 4A

C-digit-Blot-liver-ACC

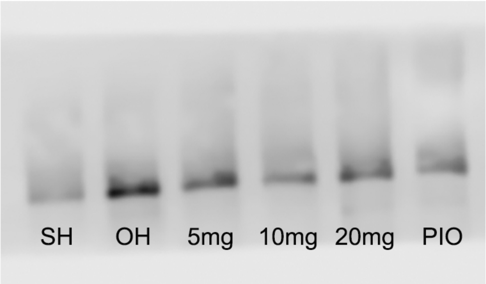

C-digit-Blot-liver-pACC-Ser79

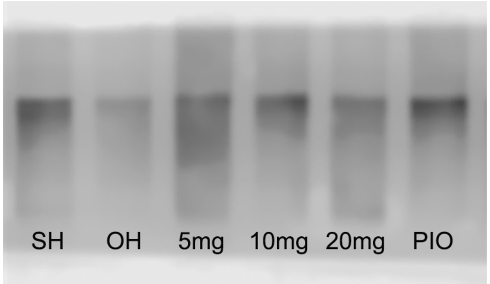

C-digit-Blot-liver-FAS

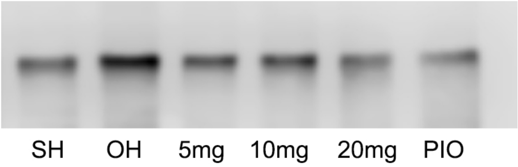

C-digit-Blot-liver-GAPDH

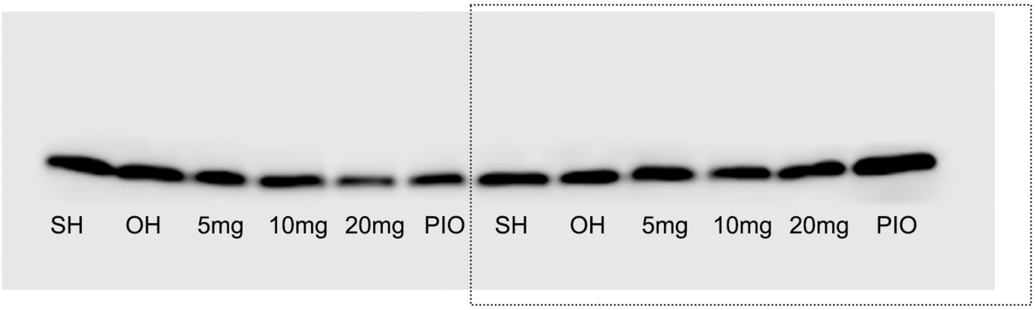

Supplementary Figure S3

Non-processed Western blot images shown in Figure 4F
The dot box outlines the area presented in the Figure 4F

C-digit-Blot-liver-CPT-1

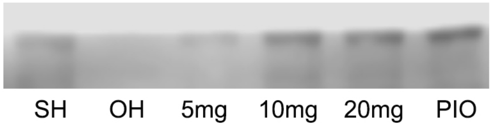

C-digit-Blot-liver-GAPDH

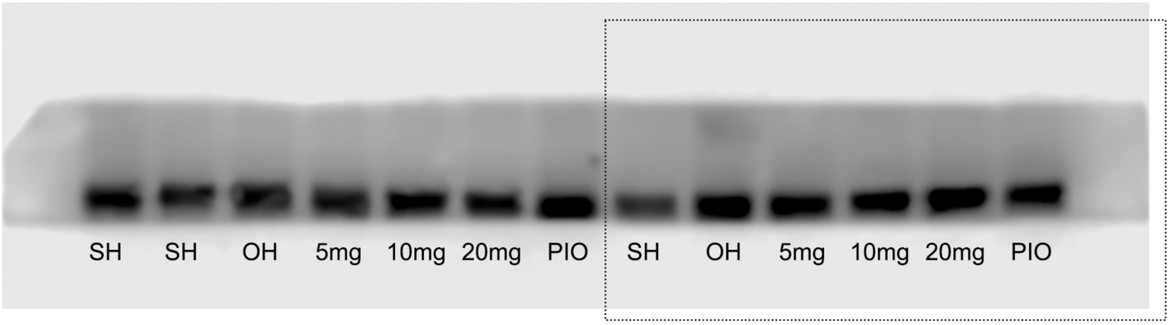

Supplementary Figure S4

Non-processed Western blot images shown in Figure 5A

C-digit-Blot-adipose-AMPK

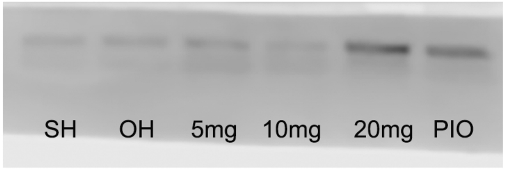

C-digit-Blot-adipose-pAMPK-Thr172

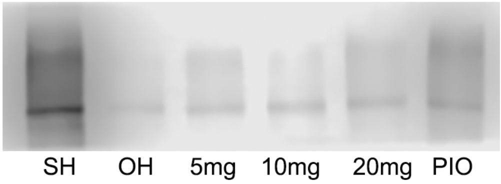

C-digit-Blot-adipose-GAPDH

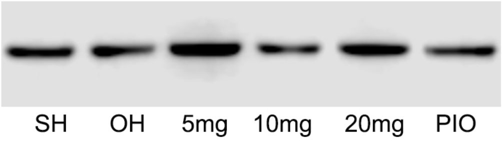

Supplementary Figure S5

Non-processed Western blot images shown in Figure 6A

The dot boxes outline the areas presented in the Figure 6A

C-digit-Blot-adipose-ACC

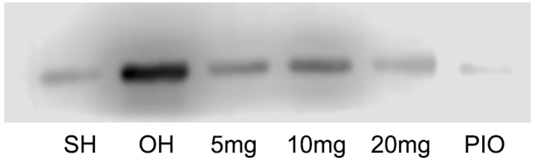

C-digit-Blot-adipose-pACC-Ser79

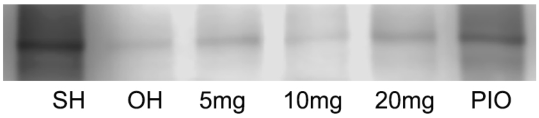

C-digit-Blot-adipose-FAS

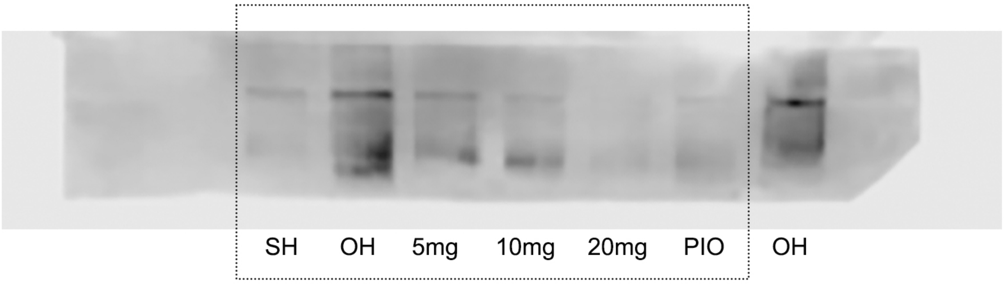

C-digit-Blot-adipose-GAPDH

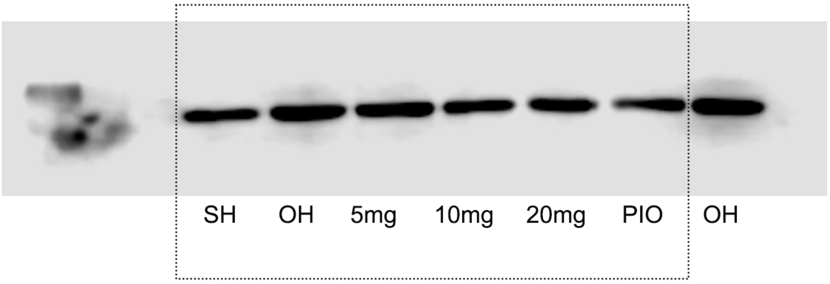

Supplementary Figure S6

Non-processed Western blot images shown in Figure 6F

C-digit-Blot-adipose-SREBP-1C

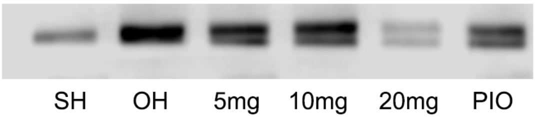

C-digit-Blot-adipose-GAPDH

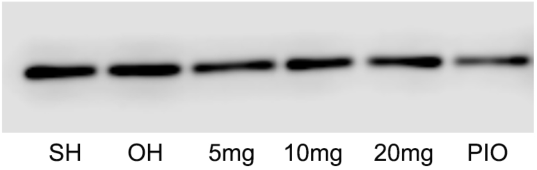

Supplement: Supplementary file 1 [file biomedicines-11-02071-s001.zip › biomedicines-2459612-supplementary.pdf]
